# Evaluating the Microbial Safety of Heat-Treated Fecal Sludge for Black Soldier Fly Larvae Production in South Africa

**DOI:** 10.1089/ees.2020.0272

**Published:** 2021-05-24

**Authors:** Daniela A. Peguero, Ellen T. Mutsakatira, Christopher A. Buckley, Gary L. Foutch, Heather N. Bischel

**Affiliations:** ^1^ Department of Civil and Environmental Engineering, University of California Davis, Davis, California, USA.; ^2^ School of Engineering, University of KwaZulu-Natal, Durban, South Africa.; ^3^ Computing and Engineering, University of Missouri Kansas City, Kansas City, Missouri, USA.

**Keywords:** fecal sludge management, heat treatment, *Hermetia illucens*, microbial inactivation, sanitation, South Africa

## Abstract

Incorporation of black soldier fly larvae (BSFL) in fecal sludge management shows promise as a resource recovery strategy. BSFL efficiently convert organic waste into valuable lipids and protein, which can be further processed into commercial products. Ensuring the microbial safety of waste-derived products is critical to the success of resource-oriented sanitation and requires the development of effective sludge treatment. This study evaluates the microbial treatment efficacy of the viscous heater (VH) for fecal sludge management and potential application of the VH in BSFL production. The VH is a heat-based fecal sludge treatment technology that harnesses the viscosity of fecal sludge to achieve pasteurization temperatures. Inactivation of *in situ Escherichia coli*, total coliform, heterotrophic bacteria, and somatic coliphage was evaluated in fecal sludge that was treated for 1–6 min at VH temperature set-points of 60°C and 80°C. The VH inactivated *in situ E. coli*, total coliform, and somatic coliphage in fecal sludge to below the limits of detection (1- to 5-log_10_ inactivation) when operated at the 80°C set-point with a 1-min residence time. Both temperature set-points achieved 1- to 3-log_10_ inactivation of *in situ* heterotrophic bacteria. The VH was also evaluated as a potential pretreatment step in BSFL production. BSFL grown in untreated and VH-treated fecal sludge demonstrated similar results, indicating little impact on the BSFL growth potential by VH-treatment. However, BSFL bioconversion rates were low for both substrates (1.6% ± 0.6% for untreated sludge and 2.1 ± 0.4 VH-treated fecal sludge).

## Introduction

Lack of access to sanitation for 2.1 billion people contributes to the spread of diarrheal disease and associated health risks (United Nations Children's Fund [UNICEF] and World Health Organization, 2019). Management of human excreta through containment, treatment, and disposal or reuse is critical to protect public health (World Health Organization, 2017). While pit latrines are commonly used for human excreta containment, untreated human excreta is often left in place or dumped into the environment rather than treated or reused (Orner and Mihelcic, 2018). Emerging fecal sludge management strategies are incentivizing treatment and reuse by transforming fecal sludge into value-added products (Strande *et al.*, 2014). Application of innovative resource-recovery technologies can enable sanitation-based entrepreneurship (Diener *et al.*, 2014).

One approach for resource-recovery incorporates black soldier fly larvae (BSFL) into the sanitation service chain for fecal sludge treatment and reuse (Gold *et al.*, 2018). BSFL can be reared on a wide variety of organic wastes, including human excreta. BSFL have high protein (40.8% ± 3.8%) and fat (28.6% ± 8.6%) content (Zheng *et al.*, 2012; Wang and Shelomi, 2017), which can be used to produce commercial products such as animal feed or biodiesel. In Durban, South Africa, the BioCycle is developing a sanitation business model based on this concept and working in collaboration with the eThekwini Water and Sanitation (EWS) unit of the local municipality to do so. The business model entails rearing BSFL on fecal sludge collected from urine diversion toilets in rural communities within the municipality, with the resulting insect biomass further processed and sold (Grau and Alcock, 2019).

Ensuring worker safety while handling fecal sludge in a BSFL production process remains a challenge. Manual labor is involved in recovering BSFL from untreated fecal sludge, and BSFL digestion processes provide limited treatment of pathogens in fecal waste (Lalander *et al.*, 2013). Rapid fecal sludge treatment technologies are needed to reduce microbial hazards associated with handling untreated fecal sludge during BSFL production. In this study, we test the efficacy of heat treatment of fecal sludge in a BSFL production pipeline. We then evaluate the impact of heat treatment of fecal sludge on BSFL growth potential and waste reduction (WR) efficiency.

**FIG. 1.**
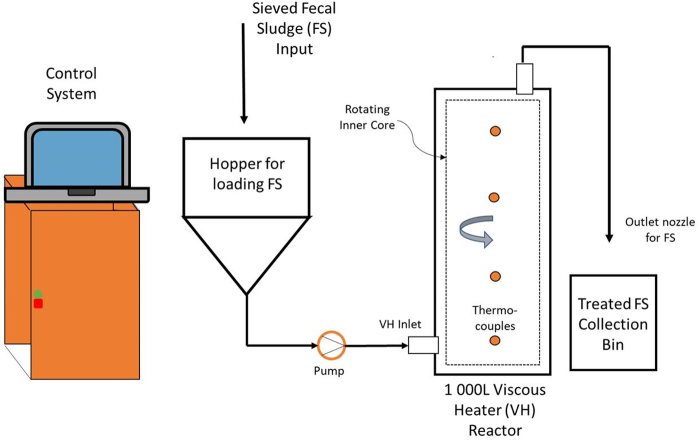
Schematic of the VH housed at the BioCycle facility located at the Isipingo. VH, viscous heater.

Pilot-scale experiments applied a new fecal sludge treatment technology, the viscous heater (VH) (Fig. 1) at different operating conditions. The VH utilizes the viscosity of input fecal sludge to generate heat and high temperatures (>60°C) (Belcher *et al.*, 2015). In the flow-through reactor, sludge passes through a small annular gap of ∼10 mm between a stationary shell and rotating inner core, which is powered by a motor. High temperature is achieved by molecular friction within the sludge. The VH requires a high-viscosity “paste” consistency of the sludge to generate sufficient heat for microbial inactivation. Prior research investigated the efficacy of VH-treatment for helminth inactivation (Belcher *et al.*, 2015), but the inactivation efficiency for bacterial and viral pathogens or pathogen surrogates has not been evaluated.

Beyond microbial inactivation during VH-treatment, the potential for microbial regrowth in fecal sludge following heat treatment may also present a challenge for BSFL production. For pathogens that grow in the environment, nutrient-rich conditions in fecal sludge may facilitate regrowth even after pretreatment at high temperatures. Microbial dynamics in fecal sludge may also influence BSFL growth potential (Zheng *et al.*, 2012). Competition for nutrients between BSFL and microorganisms in the substrate could reduce BSFL yield (Palma *et al.*, 2018). Conversely, microbial digestion of substrate may enhance the suitability of fecal sludge for BSFL production (Gold *et al.*, 2018). To evaluate the potential of the VH to inactivate enteric bacteria and viruses in fecal sludge and prevent microbial regrowth, we measured *in situ* heterotrophic plate count (HPC) bacteria, total coliform, *Escherichia coli*, and somatic coliphage levels in fecal sludge during VH-treatment and subsequent BSFL growth trials. To evaluate the impact of VH-treatment on the suitability of fecal sludge as BSFL substrate, we assessed BSFL growth and WR potentials on VH-treated and untreated fecal sludge. We use these experiments to establish operational criteria for the integration of the VH into the BSFL process.

## Methods and Materials

### Source of fecal sludge

**FIG. 2.**
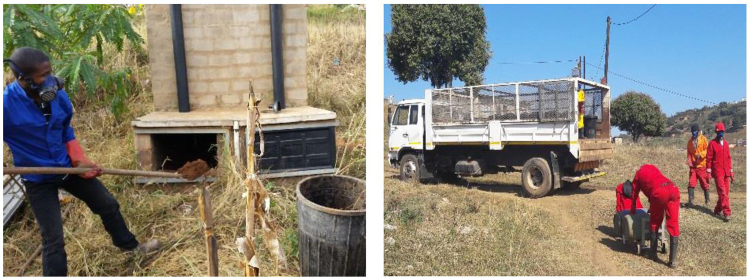
Emptying and transporting of UDT sludge (Mercer and Buckley, 2019). UDT, urine-diversion toilet.

Fecal sludge used in this study was delivered to the BioCycle facility and collected from urine-diversion toilet (UDT) vaults within a 40 km radius from the Isipingo Wastewater Treatment Works in the eThekwini Municipality, Durban, South Africa (Grau and Alcock, 2019). EWS teams manually collected sludge from vaults—both standing and active—using shovels, forks, and rakes. The municipality selected manual emptying of VIP toilets due to the challenging terrain and housing density (Wilson and Harrison, 2012). The same technique was employed to empty the UDT vaults (Fig. 2), as similar challenges with emptying VIPs were encountered with UDTs (Roma *et al.*, 2013). The sludge age ranged from ∼1 to 5 years (depending on the date of toilet installation). The municipality currently provides emptying service to all households every 2 years, but the municipality's previous policy required households to assume responsibility for emptying.

The operation of UDTs requires that users add cover material (e.g., sand, soil, or ash) as an absorbent after each use of the vault (Austin and Cloete, 2008). In the eThekwini municipality, sandy soil is typically used (Austin, 2006). Further information on the urine diversion and fecal sludge systems managed by EWS have been described elsewhere (Gounden *et al.*, 2006; Buckley *et al.*, 2008a, 2008b). Sludge was sieved to <5 mm and mixed with water to make a slurry with ∼65% moisture content, as previously described (Peguero *et al*., 2018). The sludge was further characterized and processed for experiments as described below. Research ethics review and approval was conducted by the Biomedical Research Ethics Committee (CREC) at the University of KwaZulu-Natal (BREC Ref No: BE614/17).

### Chemical characterization

Ammonia, chemical oxygen demand, total solids, and moisture content were measured in the fecal sludge before and after VH treatment experiments according to Standard Methods (Eaton *et al.*, 2005). Fecal sludge used for BSFL growth tests was characterized for its moisture, carbon, and nitrogen contents. For carbon and nitrogen analysis, 0.2 ± 0.1 g fecal sludge samples were homogenized using a stirring rod in a ceramic crucible. Carbon and nitrogen content in the samples were determined using an automated elemental CNS analyzer (Leco TruMac CNS Macro Analyzer). Macro sample combustion was achieved using a pure oxygen environment at high temperatures (1350°C) in a ceramic horizontal furnace. Crude protein content in the fecal sludge was estimated from the nitrogen content using a conversion factor of 6.25 (Jones, 1931). Samples for nitrogen and carbon were analyzed in triplicate.

### VH treatment of fecal sludge

Tests were conducted using a pilot-scale VH (1,000 L/h) operated at the BioCycle facility at the Isipingo Wastewater Treatment Works in Durban, South Africa. Microbial inactivation in the fecal sludge was evaluated at two temperature set-points (60°C and 80°C) and five or six different reactor residence times (1–6 min). Three factors influence the VH operating temperature: (1) input sludge viscosity, (2) speed of the rotating inner core, and (3) the influent flow rate. The influent flow rate was controlled using a computation fluid dynamics model and program (German *et al.*, 2018) while the rotational speed of the inner core was manually adjusted to compensate for changes in fecal sludge viscosity. Temperature was monitored using four thermocouples installed along the height of the reactor (Peguero *et al.*, 2018). Each temperature set-point was tested in duplicate for a total of four VH-treatment trials. For each temperature trial, untreated fecal sludge samples were collected at the beginning of the trial, and VH-treated samples were collected after each residence time. All samples were processed immediately after VH-treatment for measurement of *E. coli*, total coliform, HPC bacteria, and somatic coliphage concentrations.

### BSFL growth on fecal sludge

The experimental setup for 13-day BSFL growth trials consisted of four test crates: two containing VH-treated fecal sludge and two containing untreated fecal sludge. Four identical control crates were prepared without the addition of BSFL neonates. VH-treated fecal sludge was prepared within 24 h before use in the experiment using an 80°C set-point and a 5-min residence time in the VH. BSFL neonates were grown on custom formulated feed for 4 days before augmentation in the crates. BSFL neonates and feed were generously provided by AgriProtein (Cape Town, South Africa). Larvae were transferred with remaining formulated feed (2.16 kg) to crates that each contained 20.6 ± 3.5 kg of VH-treated or untreated fecal sludge. Approximately 22,000 BSFL (130 ± 30 g of larvae) were transferred to each test crate. The initial mass of fecal sludge used in experiments assumed a feeding rate of 70 mg of fecal sludge larvae^−1^ day^−1^ over a 13-day growth period (Mutsakatira *et al.*, 2018). BSFL development and microbial concentrations in the fecal sludge were evaluated at seven time points during the 13 days. At each time point, 30 larvae were taken from each crate, rinsed with tap water, transferred to small, dry weighboats, and weighed to determine the composite wet weight. At the start and end of the experiment, composite larvae and fecal sludge dry weights were measured after 24 h drying at 105°C. For microbial analysis, 5 g of fecal sludge was sampled from each container.

### Biomass conversion and WR calculations

The bioconversion rate (BCR) is calculated from the ratio of the dry weight (*dw*) of the larval mass gained to the *dw* of the initial waste substrate:


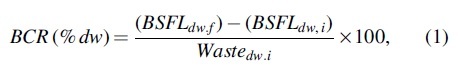


where *BSFL_dw,i_* and *BSFL_dw,f_*, are the total initial and final mass of the *BSFL* (g dw larvae), and *Waste_dw,i_* is the initial dry mass (g dw waste) of the VH-treated or untreated fecal sludge along with the remaining formulated feed that was used at the start of the experiment.

The WR rate represents overall substrate degradation (Diener *et al.*, 2009; Lalander *et al.*, 2014) and was calculated as follows:


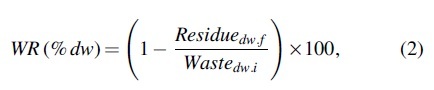


where *Residue_dw,f_* is the dry weight of substrate remaining at the end of the experiment (g dw residue).

### Microbial assays

For heterotrophic bacteria, *E. coli* and total coliform analysis, fecal sludge samples were serially diluted in phosphate-buffered saline (PBS; 2 mM MgCl_2_, 0.3 mM KH_2_PO_4_, pH 7.2). About 1 mL of each of six dilutions was plated on 3M™ Petrifilm™ dryplates (3M Company, Sandton, South Africa). The plates were incubated according to manufacturer protocol at 37–40°C for 24 h for total coliform and *E. coli* and 48 h for HPC. Somatic coliphage (viruses) were measured in PBS-diluted fecal sludge samples using the double agar layer method with *E. coli* (ATCC 13706) as the cultured host strain. The plates were incubated at 37°C overnight alongside the positive control and a blank containing only *E. coli*. Bacteriophage φX174 (ATCC 13706-B1) served as a positive control for somatic coliphage (Eaton *et al.*, 2005).

## Results and Discussion

### Heat treatment of fecal sludge

Results from the two-temperature set-points of 60°C and 80°C selected for VH experiments provide information for two application scenarios. The higher temperature setting of 80°C was selected as a conservative temperature to achieve microbial inactivation and was expected to demonstrate complete inactivation of enteric bacteria and viruses. Previous studies using a laboratory-scale VH to treat ventilated improved pit latrine fecal sludge indicated 90% inactivation of soil-transmitted helminth eggs when the fecal sludge was treated at or above 70°C (Belcher *et al.*, 2015). The lower temperature setting was selected to evaluate the microbial treatment efficacy of the VH operated under energy-saving conditions. The full temperature profiles were previously reported (Peguero *et al.*, 2018) and indicated relatively stable operating temperatures for short reactor residence times, while temperatures were less stable over longer reactor residence times. The average operating temperature throughout the reactor for the first trial of the 1-min residence time of 60°C and 80°C set-point experiments was 62.8°C and 83.4°C, respectively.

#### Inactivation of fecal indicator bacteria and viruses

Fecal indicator bacteria and somatic coliphage were recovered in all untreated fecal sludge samples, which had an overall average moisture content of 65.8 (± 10.9) %. Since target organisms were not inoculated into the fecal sludge, initial *in situ* concentrations varied with each batch test of residence time. *In situ* concentrations of *E. coli* and total coliform ranged from 4.3 × 10^3^ to 2.1 × 10^5^ CFU/g of wet fecal sludge, whereas, concentrations of somatic coliphage were typically two to three orders of magnitude lower, ranging from 3.1 × 10^1^ to 1.5 × 10^2^ PFU/g of wet fecal sludge. Trial 4 was an exception, where initial concentrations of somatic coliphage were measured at 2.87 (± 0.73) × 10^4^ PFU/g of wet fecal sludge. We report log_10_- reduction for each of the test organisms based on average initial concentrations in fecal sludge measured on the same day.

**FIG. 3.**
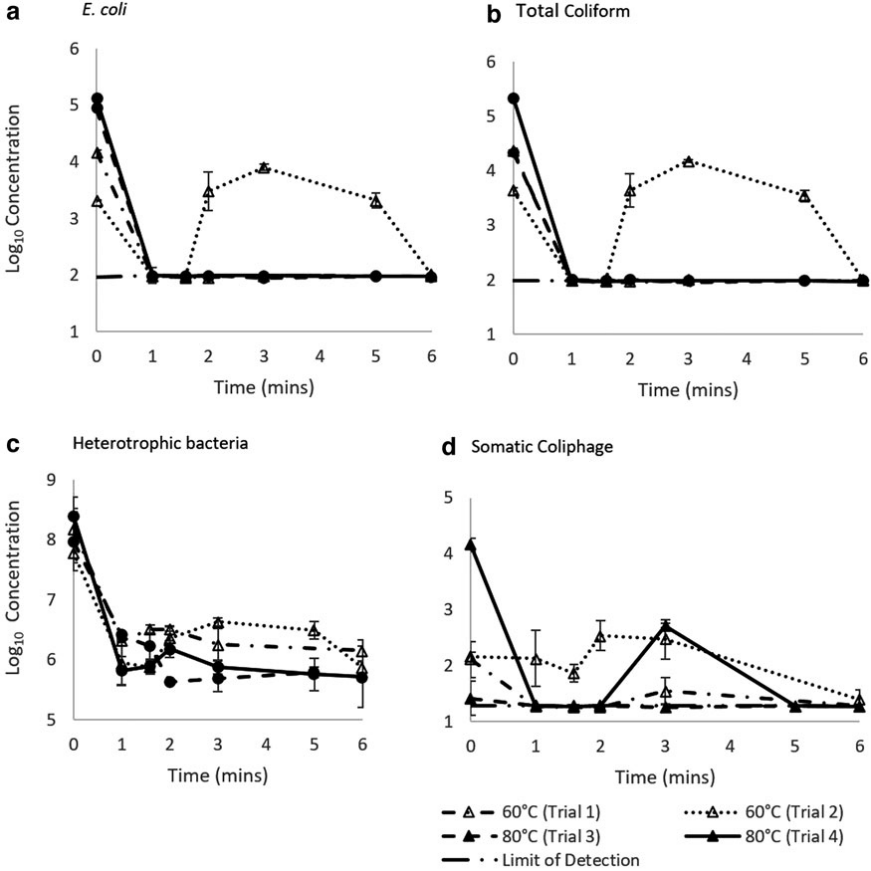
Log_10_ concentration of *in situ* **(a)** *Escherichia coli* (CFU/g), **(b)** total coliform (CFU/g), **(c)** heterotrophic bacteria (CFU/g), and **(d)** somatic coliphage (PFU/g) in fecal sludge after heat treatment at 60°C (Δ) or 80°C (●). Four batches of sludge were treated (Trials 1 to 4). Results are shown as log_10_ concentration plots with the nondetect results displayed at the limit of detection. Error bars represent the standard deviation of triplicate measurements (*n* = 3). CFU, colony forming unit; PFU, plaque forming unit.

Fecal indicator bacteria were generally more sensitive to heat treatment than viruses, especially at the lower temperature treatment condition. For example, a 2-log_10_ reduction of *E. coli* and total coliform was observed during the first of two trials at the 60°C set-point at a 1-min residence time (Fig. 3). At this same setting, a 1-log_10_ reduction of somatic coliphage was achieved (Fig. 3). Variability in survival of test organisms was observed during Trial 2 conducted at 60°C, but 2-log_10_ inactivation of *E. coli* was nevertheless achieved at the 1- and 6-min residence times. In contrast, somatic coliphage were generally more resistant than *E. coli* to inactivation at the 60°C set-point. While heat treatment at the lower temperature set-point of 60°C may provide sufficient inactivation of *E. coli* and total coliform, viruses may persist at this condition. Results were consistent with previous observations: Berg and Berman (1980) found that *E. coli* and total coliform are 9 to 10 times more sensitive to thermophilic temperatures than viruses in fecal sludge digesters.

The 80°C set-point with 1-min residence time was the most effective treatment condition, as a 3-log_10_ reduction in *E. coli* and total coliform was observed. Virus inactivation followed a similar pattern to the fecal indicator bacteria. Approximately 3-log_10_ inactivation of somatic coliphage was observed during the fourth trial at the 80°C set-point with 1-min residence time (Fig. 3). Initial concentrations of somatic coliphage in Trial 4 were higher than on other experimental days, so the log reduction was greater. During the 3-min residence time at the 80°C set-point (Trial 4), the temperature dropped to 70°C and only 1.5-log_10_ inactivation of somatic coliphage was observed. At 70°C, the fecal indicator bacteria were still fully inactivated (Fig. 3). To ensure complete inactivation of both fecal indicator bacteria and somatic coliphage during VH-treatment, reactor operators should ensure the temperatures reach and maintain the 80°C set-point.

During both trials of the 80°C set-point, efficient inactivation of fecal indicator bacteria and somatic coliphage was observed for the 1-min residence time, without additional benefit of longer residence times. Gantzer *et al.* (2001) found thermophilic treatments of sludge (aerobic digestion and heat) resulted consistently in a 3.5-log_10_ reduction of *E. coli* at 60°C for a minimum of 10-h. The VH achieved similar results in 1-min of treatment at 80°C. The drastically reduced treatment time would be highly beneficial for treating large volumes of fecal sludge. Nevertheless, heterotrophic bacteria were detected above the detection limit [9.4 (± 0.3) × 10^2^ CFU/g wet fecal sludge] after treatment for all treatment residence times evaluated (Fig. 3), indicating sterilization of fecal sludge was not achieved (Peguero *et al.*, 2018). Initial levels of heterotrophic bacteria ranged from 1 × 10^7^ to 2.3 × 10^8^ CFU/g of wet fecal sludge and were detected in all untreated fecal sludge samples. Approximately 1.5-log_10_ to 2.5-log_10_ inactivation of heterotrophic bacteria was observed at both treatment temperatures. Contrary to expectation, a longer residence time did not result in an increase in inactivation of heterotrophic bacteria (Peguero *et al.*, 2018).

### BSFL development trials in untreated and VH-treated fecal sludge

The overall aim of the BSFL growth trials was to evaluate the potential application of heat treatment of fecal sludge before BSFL growth. We hypothesized that larvae grown in VH-treated sludge would outperform untreated sludge due to increased nutrient availability, homogenization of the sludge that the VH provides, reduced substrate particle size, and removal of detritus. BSFL development was evaluated in untreated and VH-treated fecal sludge to test this hypothesis. We also hypothesized that the presence of BSFL would promote regrowth of fecal indicator organisms, by mixing and aeration of the sludge. *In situ E. coli*, total coliforms, HPC bacteria, and somatic coliphage were monitored in untreated and VH-treated fecal sludge during BSFL development to evaluate this hypothesis.

#### BSFL growth and substrate characterization

The bioconversion and WR rates by larvae were similar for untreated and VH-treated fecal sludge (Table 1), suggesting that VH-treatment of fecal sludge did not significantly affect the potential to use fecal sludge as a larval substrate. Nevertheless, the low BCRs (1.6% ± 0.6% for untreated sludge and 2.1 ± 0.4 VH-treated fecal sludge) indicate that BSFL were not effective in reducing fecal sludge mass, even with the additional mixture of 2.16 kg of the custom formulated feed at the outset of the experiment. A higher BCR of 22.9% was obtained for BSFL grown on fresh feces (Banks *et al.*, 2014). Larvae mass production was correspondingly low (Fig. 3). The mean larval mass after 13 days of growth in untreated sludge (72.6 ± 6.2 mg) or VH-treated fecal sludge (69.6 ± 0.5 mg) were lower than larvae reared on fresh human feces for 12 days (298.6 ± 3.9 mg) (Banks *et al.*, 2014).

**Table 1.** Efficiency of the Larvae Consuming the Viscous Heater-Treated Fecal Sludge and the Untreated Fecal Sludge

	BCR (%)	WR (%)	C:N ratio
	Average ± range (*n* = 2)		
Untreated residue	1.6 ± 0.6	9.4 ± 0.9	11.0 ± 1.9
VH treated residue	2.1 ± 0.4	7.1 ± 1.1	12.5 ± 0.5

BCR, bioconversion rate; VH, viscous heater; WR, waste reduction.

Substrate composition is a major factor in BSFL growth. Low C:N ratios in the fecal sludge, and correspondingly low sludge protein content, provided poor growth conditions. Protein is critical for larval growth (Wang and Shelomi, 2017), but fecal sludge from pit latrines typically has low nutritional quality. The initial protein content in the fecal sludge was calculated to be 8% ± 1%, less than half that in restaurant waste (Cammack and Tomberlin, 2017). C:N ratios for untreated and VH-treated fecal sludge were 11.0 ± 1.9 and 12.5 ± 0.5, respectively. For comparison, BSFL were grown successfully on dairy manure and chicken manure with C:N ratios of 21.77 ± 1.32 and 15.69 ± 2.07, respectively (Rehman *et al.*, 2017). An optimal C:N ratio of 30 was determined for BSFL growth on almond hulls (Palma *et al.*, 2018). For fecal sludge, the addition of other organic waste substrates with higher levels of carbon, such as vegetable or fruit waste, to increase the C:N ratio may aid in sludge mass reduction and enhance growth of BSFL.

The form of nitrogen may also influence BSFL growth efficiency. Nitrogen in sludge can be present in organic (e.g., amino acids and amines) or inorganic (e.g., ammonia, nitrite, and nitrate) forms (Strande *et al.*, 2014). Total Kjeldahl nitrogen, which includes organic and ammonia/ammonium nitrogen, was 30 mg/g dry on average in sludge from urine diversion systems in Durban (Strande *et al.*, 2014). We expected VH treatment of sludge could release nutrients (Case and Jensen, 2019), increasing available nitrogen concentrations for larvae growth in the sludge (Gold *et al.*, 2018). Average ammonia concentrations essentially doubled following VH treatment (Table 2), but there was wide variability and the change was not significant (Student's *t*-test, *p* = 0.30). Heat treatment may have alternatively increased nutrient accessibility for microorganisms, which could preferentially utilize nitrogen over the larvae (Palma *et al.*, 2018). As demonstrated by HPC bacteria levels in the fecal sludge, the microbial communities were still active following VH-treatment at the 80°C set-point. Evaluation of BSFL growth after fecal sludge sterilization would provide further insight into the role of substrate microbial dynamics on BSFL growth.

**Table 2.** Results of Characterization of the Untreated Urine-Diversion Fecal Sludge Before and After 1-Min of Viscous Heater Heat Treatment Before Conducting Black Soldier Fly Larvae Trials

	Untreated	60°C	80°C
	Average ± standard deviation		
TS (%)	34 ± 11	23.0 ± 0.1	30.5 ± 8.3
Moisture content (%)	66 ± 11	77 ± 16	70 ± 8
Ammonia (NH_3_-N mg/g dry FS)	3.0 ± 1.7	6.5 ± 3.5	5.7 ± 5.5
COD (mg/g dry FS)	330 ± 120	480 ± 140	490 ± 170

Average results are shown untreated (*n* = 5), 60°C treatment (*n* = 2) and 80°C treatment (*n* = 3) trials.

COD, chemical oxygen demand; TS, total solids.

**FIG. 4.**
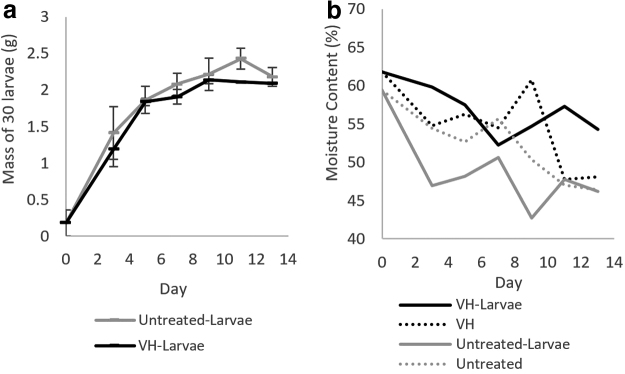
**(a)** BSFL growth in VH-treated and untreated fecal sludge. The average of measurements from two growth experiments is shown with the range as error bars (*n* = 2). **(b)** Moisture content in the VH-treated (*n* = 6) and untreated fecal sludge (*n* = 6) with or without larvae. The average moisture content of two reactors for each condition is presented. BSFL, black soldier fly larvae.

Confounding effects of several additional experimental conditions on BSFL growth require further evaluation. First, substrate moisture content can influence microbial levels in the substrate, altering competition between BSFL and microorganisms for nutrients. The moisture content in the VH-treated fecal sludge with larvae was consistently higher than untreated fecal sludge (Fig. 4). Increasing substrate moisture content improved larvae growth on almond hulls (Palma *et al.*, 2018). Second, the ambient temperature will impact BSFL growth. BSFL are native to warmer climates and thrive in temperatures between 23°C and 31°C (Liu *et al.*, 2008). Ambient temperature over the course of our experiments dipped below this range, oscillating between 14.55°C and 34.78°C. Third, the larvae development time will impact growth. Allowing for longer development time on the sludge could increase larvae weight. Finally, particle size in the substrate may impact BSFL growth. Wormlions (*Vermileonidae*) demonstrated a preference for finer particle sizes (≤110 μm) (Devetak, 2008). Particle size was not characterized in the fecal sludge due to analytical limitations.

#### Microbial dynamics in fecal sludge during BSFL development: Heterotrophic bacteria

**FIG. 5.**
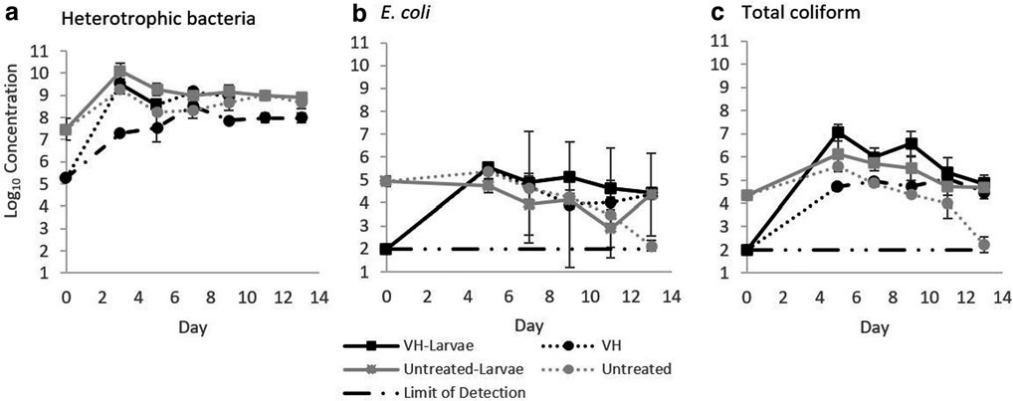
Log_10_ concentrations of **(a)** heterotrophic bacteria (CFU/g), **(b)** *Escherichia coli* (CFU/g), and **(c)** total coliform (CFU/g) in fecal sludge stored with BSFL or without BSFL over 13 days. Fecal sludge was obtained from UDTs, screened to remove large debris, and either heat-treated at 80°C for 5 min using the VH or left untreated before the experiment. Results from reactors with VH-treated fecal sludge (*n* = 4) are shown in *black*, and results from reactors with untreated fecal sludge are shown in *gray* (*n* = 4). Results from reactors that contained BSFL are shown with (□) *square labels* and *solid lines*. Results from reactors without BSFL are shown with (○) *circle labels* and *dotted lines*. Controls consisted of treated or untreated sludge stored without BSFL or additional feed. Only initial *E. coli* and total coliform concentrations in VH-treated sludge were below the limit of detection (shown at the limit of detection).

Initial concentrations of HPC in untreated fecal sludge acquired for the pilot-scale BSFL growth trials were 4.66 ± (3.29) × 10^7^ CFU/g wet fecal sludge. After 5-min heat treatment in the VH at an 80°C set-point, HPC were 2-log_10_ lower in the VH-treated sludge than in untreated sludge. HPC bacteria in the VH-treated sludge subsequently increased during the experiment and stabilized around 9- to 10-log_10_ CFU/g wet fecal sludge in microcosms with or without BSFL (Fig. 5). Results were consistent with the findings observed at the laboratory scale (data not shown), where no inactivation of HPC bacteria was observed in fecal sludge during BSFL development or fecal sludge controls without BSFL. HPC levels in VH-treated sludge did increase to slightly higher levels in the presence of BSFL when compared with VH-treated sludge without BSFL. However, the presence of BSFL did not appear to have a substantial net influence on HPC (e.g., by ingestion of bacteria as substrate) relative to the control.

#### Fecal indicator bacteria regrowth and inactivation during BSFL growth

*E. coli* and total coliform were detected in all fecal sludge samples before treatment, at 9.7 (± 5.74) × 10^4^ CFU/g wet fecal sludge and 2.14 (± 0.003) × 10^4^ CFU/g wet fecal sludge, respectively. After VH treatment, *E. coli* and total coliform levels were below the detection limits. *E. coli* and total coliform concentrations recovered in the VH-treated fecal sludge in the 4 days following treatment, both with and without BSFL present (Fig. 5). *E. coli* levels decreased somewhat during storage of the untreated fecal sludge without BSFL from day 6 to 13, and to a lesser extent in other reactors. Total coliform exhibited similar patterns to *E. coli*. Erickson *et al.* (2004) found that the presence of BSFL in chicken manure contributed to *E. coli* O157:H7 inactivation, especially at elevated temperatures (27°C or 32°C). Banks *et al.* (2014) reported 0.67-log_10_ reduction of thermotolerant coliforms in fecal sludge containing BSFL over 1 week. In the present study, inactivation of *E. coli* and total coliform were limited and did not appear to be enhanced by larval activity. Decreasing moisture content through time (Fig. 4) may have contributed to observed inactivation. Yeager and Ward (1981) found that pathogen inactivation was effective in long-term storage of fecal sludge that maintained a moisture content between 10% and 50%. The moisture content of the fecal sludge after 13 days of storage ranged from 46.4% to 54.3%.

#### Suppression of somatic coliphage during BSFL growth

**FIG. 6.**
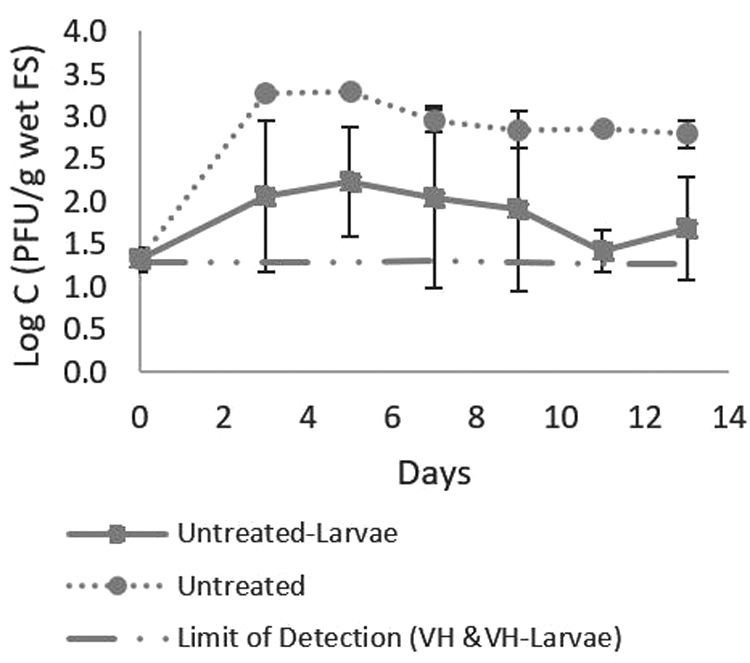
Somatic coliphage with the control (without BSFL) and experiment (with BSFL) in untreated fecal sludge (*n* = 4). Somatic coliphage in the VH-treated fecal sludge (*n* = 4) were below the detection limit in all samples (data not shown).

Somatic coliphages were detected in untreated fecal sludge from 3.13 (± 2.38) × 10^1^ to 2.87 (± 0.73) × 10^4^ PFU/g of wet fecal sludge and were no longer detected following heat treatment of fecal sludge by VH. No regrowth of somatic coliphage in the VH-treated fecal sludge stored was observed, with or without BSFL present (Fig. 6). For somatic coliphage to propagate in the environment, a bacterial host population of greater than 10^4^ CFU/mL is needed (Wiggins and Alexander, 1985). Unlike for bacteria, some suppression of somatic coliphage concentrations appeared to be linked to the presence of BSFL in untreated fecal sludge. Somatic coliphage concentrations in untreated fecal sludge with BSFL were lower, and more highly variable, than concentrations in untreated fecal sludge without BSFL (Fig. 6). Moisture content was similar in the untreated fecal sludge with BSFL (46.2% ± 4.8%) and the untreated fecal sludge without BSFL (46.4% ± 1.7%) and unlikely to contribute to the observed differences in somatic coliphage concentrations. Concentrations of ammonia, which is a microbial disinfectant (Jenkins *et al.*, 1998), were also similar in both reactors, and relatively low (3.0 ± 1.7 NH_3_-N mg/g dry fecal sludge in untreated and 5.7 ± 5.5 NH_3_-N mg/g dry fecal sludge in VH-treated). Lalander *et al.* (2013) observed a 0.64-log_10_ reduction of the φX174 bacteriophage over a week after spiking bacteriophage into sludge stored with BSFL. However, the inactivation was not significant compared with controls without BSFL. Further evaluation of the microbial inactivation potential of BSFL, and mechanisms contributing to these observations, is warranted.

Somatic coliphage in the VH-treated fecal sludge (*n* = 4) were below the detection limit in all samples (data not shown).

## Conclusion

The results of this study demonstrated that, with some added temperature controls, the VH can be used for consistent, rapid inactivation of microorganisms in fecal sludge. The VH was found to be effective for a 1- to 5-log_10_ inactivation of *E. coli*, total coliform, and somatic coliphage when operated at elevated temperatures (i.e., 80°C). Incorporation of the VH in BSFL production did not impact BSFL production and WR efficiencies, a positive indication for its use in resource recovery. However, utilizing the VH as a pretreatment step for fecal sludge in a BSFL production pipeline did not prevent regrowth of *E. coli* and total coliform during BSFL development. While other studies have indicated that the presence of BSFL may lead to the inactivation of microbes in the substrate, the present study did not observe this phenomenon conclusively. Evaluation of higher temperature VH-treatment (e.g., 100°C) of sludge used for BSFL growth is needed to assess the potential for improved microbial safety and suppression of *E. coli* regrowth. The addition of nutritional substrates to fecal sludge and longer growth times should be evaluated for improved BSFL development when using UD sludge as substrate.
